# A novel chrysovirus from a clinical isolate of *Aspergillus thermomutatus* affects sporulation

**DOI:** 10.1371/journal.pone.0209443

**Published:** 2018-12-20

**Authors:** Mahjoub A. Ejmal, David J. Holland, Robin M. MacDiarmid, Michael N. Pearson

**Affiliations:** 1 School of Biological Sciences, the University of Auckland, Auckland, New Zealand; 2 Infectious Diseases Unit, Division of Medicine, Staff Centre, Middlemore Hospital, Auckland, New Zealand; 3 The New Zealand Institute for Plant and Food Research Limited, Auckland, New Zealand; Leibniz-Institut fur Naturstoff-Forschung und Infektionsbiologie eV Hans-Knoll-Institut, GERMANY

## Abstract

A clinical isolate of *Aspergillus thermomutatus* (Teleomorph: *Neosartorya pseudofischeri*) was found to contain ~35 nm isometric virus-like particles associated with four double-stranded (ds) RNA segments, each of which coded for a single open reading frame. The longest dsRNA element (3589 nt) encodes a putative RNA-dependent RNA polymerase (1114 aa), the second longest dsRNA element (2772 nt) encodes a coat protein (825 aa), and the other two dsRNAs (2676 nt, 2514 nt) encode hypothetical proteins of 768 aa and 711 aa, respectively. Phylogenetic analysis of the amino acid sequences showed 41–60% similarity to the proteins coded by the dsRNAs of the most closely related virus, *Penicillium janczewskii chrysovirus 2*, indicating that it is a new species based on the International Committee on Taxonomy of Viruses criteria for the genus *Chrysovirus*. This is the first virus reported from *A*. *thermomutatus* and was tentatively named *Aspergillus thermomutatus chrysovirus* 1. A virus free line of the fungal isolate, cured by cycloheximide treatment, produced large numbers of conidia but no ascospores at both 20°C and 37°C, whereas the virus infected line produced ten-fold fewer conidia at 20°C and a large number of ascospores at both temperatures. The effects of the virus on fungal sporulation have interesting implications for the spread of the fungus and possible use of the virus as a biological control agent.

## Introduction

The vast majority of the reported mycoviruses contain double-stranded (ds) RNA genomes; a lesser number have single-stranded (ss) RNA genomes, while only a few mycoviruses possess single-stranded DNA [1]. Consequently dsRNAs are often the template of choice when investigating RNA viruses, as they represent either the genome or replicative form of most mycoviruses and are more stable and easier to purify than single-stranded (ss) RNA [2]. Many *Aspergillus* species have been reported to be infected with mycoviruses, or uncharacterised dsRNA segments [3]. These include *Aspergillus* section *Nigri* [4, 5, 6], *Aspergillus* section *Flavi* [7], *Aspergillus* section *Circundati*, and section *Fumigati* [8], *Aspergillus* section *Clavati* [9], *A*. *foetidus* [10, 11], *A*. *niger* [12], *A*. *flavus* [13, 14, 15], *A*. *fumigates* [16, 17, 18, 19, 20] and *A*. *ochraceus* [21, 22], with an incidence of 7–50% in all examined species [3].

*Aspergillus thermomutatus* (Paden) S. W. Peterson (Teleomorph: *Neosartorya pseudofischeri* S. W. Peterson) was first described by Paden [23] and re-examined by Peterson [24]. It is known to occur in soil and to be temperature tolerant, being able to grow at 50°C [25, 26]. Although the species is not commonly known as a human pathogen [27], there are several reports of it causing human infections in immunocompromised patients [27, 28, 29]. Mycoviruses are believed to rarely occur in sexually reproducing *Aspergilli* [3] and there are no previous reports of mycoviruses infecting *A*. *thermomutatus*. However, *A*. *thermomutatus* often produces both anamorph (asexual) and teleomorph (sexual) stages together in the same culture [30], and a clinical isolate showing noticeable changes in colony texture, sector formation and fluctuations in sporulation was found to contain dsRNAs indicative of virus infection. Mycoviruses have shown potential as biological control agents for plant pathogenic fungi, which raises the question whether they might also be used to help to treat human fungal diseases. A major attraction of mycoviruses as biocontrol agents is their high degree of specificity to fungi, and therefore their promise of safety for potential use in humans. The use of mycoviruses as a tool to combat invasive fungal infection in animals and humans has not yet been explored in detail [31].

## Materials and methods

### Fungal origin and maintenance

An isolate of *A*. *thermomutatus* (Ath1) was obtained on 10/7/2012 from the fungal culture collection at Middlemore Hospital, Auckland, New Zealand. While the fungal cultures studied were originally derived from clinical isolates their origin was anonymous and the material analysed consisted of pure fungal cultures that contained no human material. Cultures were maintained on Potato Dextrose Agar (PDA, Difco) at 37°C in the dark. The Ath1 isolate was studied in a Physical Containment Level 2 laboratory, where all culturing procedures were conducted in a biological safety cabinet under the highest possible level of decontamination.

### DsRNA extraction and electrophoresis

DsRNA was extracted from fungal mycelium and purified virus particles, according to the method of Valverde et al. [32] as modified by Khalifa and Pearson [33]. For visualization the dsRNA was mixed 5:1 with loading dye (30% glycerol, 1 X tris-borate-EDTA-buffer (TBE), 2% ficoll-400, 0.25% xylene cyanol, 0.25% bromophenol blue) and the mix loaded into a 1% agarose gel prepared in 0.5X TBE buffer and pre-stained with 5 μl RedSafe nucleic acid stain (Intron) per 100 mL. The gel was run in 0.5 X TBE buffer at 90 V for ~ 45 min and visualised using a UV Gel Doc XR+ system (BIO RAD). The size of the dsRNA bands was estimated against a 1 kb plus DNA ladder (Invitrogen). The dsRNA nature of the bands was confirmed as described by Howitt et al. [34].

### Virus purification

Virus-infected Ath1 was grown in conical flasks containing 200 mL Yeast Extract Peptone Dextrose broth (YPD) and incubated on a shaking incubator at 180 rpm in the dark. Approximately 10 g of fungal mycelium was harvested on a filter paper using vacuum filtration, ground to a fine powder in liquid nitrogen and transferred to a 50-mL Falcon tube containing 20 mL of sodium phosphate buffer (SPB: 0.1 M, pH 7.0) and 10 mL chloroform. The mixture was incubated for 30 min on ice on an orbital shaker at 230 rpm then centrifuged at 10,000 x g for 30 min at 4°C. The upper aqueous phase was centrifuged at 120,000 x g for 2 h at 4°C and the resultant pellet resuspended in 1 mL SPB (0.02 M pH 7.0) for 4 h at 4°C. The suspension was clarified by centrifugation at 10,000 x g for 20 min at 4°C, the supernatant centrifuged at 120,000 x g for 2 h at 4°C and the pellet resuspended in 0.5 mL SPB (0.02 M, pH 7.0) overnight at 4°C. Following centrifugation at 10,000 x g for 20 min a 50-μL drop of the supernatant was negatively stained with 2% uranyl acetate (pH 4.0) and observed for virus particles using a Phillips CM12 TEM. The size of 100 virus particles was determined relative to the scale bar (200 nm) incorporated into the EM image from enlarged photomicrographs (79 x 79 cm) using Poster Printer software v 3.01.43 (www.ronyasoft.com).

### Genome sequencing

#### 454 sequencing

Initial partial sequence of purified dsRNA, obtained by 454 sequencing (accession: SAMN10160075), was used as the starting point for complete genome sequencing. Preparation of samples was performed as described by Roossinck et al. [35]. A primer (5`-CTCACCTTCGGATCCTCC_12_-3`) with a unique four nucleotide index at the 5`end was used to generate dsDNA fragments with random lengths (~600–1500 bp), which were sequenced by Macrogen Inc. (South Korea) as described by Roossinck et al. [35]. Reads were *de novo* assembled in Geneious software version 8.0.3 [36].

#### Cloning and sanger sequencing

For each of the four dsRNA elements, viral dsRNA was gel purified using an Axyprep DNA gel extraction kit (AXYGEN) and used as a template for cDNA synthesis, dsDNA amplification and determination of 5`and 3`terminal sequences as described by Khalifa and Pearson [37]. PCR products were cloned in a pCR2.1-TOPO vector in One Shot DH5-T1R *Escherichia coli*-competent cells and sequenced using vector-specific primers (M13 forward 5´-GTAAAACGACGGCCAG-3´ and M13 Reverse 5´-CAGGAAACAGCTATGAC-3´). Nucleotide sequences were assembled using Geneious version 8.0.3 [36] as described by Khalifa and Pearson [37].

### Bioinformatics and phylogenetic analysis

The potential secondary structure of the 5`and 3`termini of the dsRNA1 was predicted and the minimum free energy (ΔG) was estimated using Quickfold software (http://unafold.rna.albany.edu/?q=DINAMelt/Quickfold) with the following parameters: RNA at 37°C, Na^+^ = 1 M, Mg^++^ = 0 M, sequence type = linear, distance between paired bases = no limit. The closest published sequences to the consensus sequences (for both the PCR products and 454 sequence data) were determined using BLASTn (blast.ncbi.nlm.nih.gov/Blast.cgi). Where BLASTn failed to find a similar sequence, BLASTx (translated nucleotide to protein) was used to find the closest amino acid pairwise sequence. Deduced amino acid sequences of the RNA-dependent RNA polymerases (RdRPs) of the new *A*. *thermomutatus* virus and the closest published sequences were aligned using MUSCLE multiple sequence alignment software [38] and used to generate a phylogenetic tree using the Neighbour-joining method [39] for distance calculated with a Poisson model and a gamma distribution of rates between sites with 1000 bootstrap replications using MEGA 6 [40].

### PCR test for the presence of *A*. *thermomutatus chrysovirus* 1 (AthCV1)

To test for the presence of AthCV1, total RNA was extracted from fungal cultures using a Spectrum Plant Total RNA Kit (Sigma-Aldrich), as described by the manufacturer, and used as a template for one-step RT-PCR using a virus-specific primer pair (forward primer: 5`-CGAGTGTGAGGCATCAAAGC-3`and a reverse primer: 5`-TGTCGCATGATGCATATAATTGGG-3`) and PrimeScript One-step RT-PCR Kit (Takara Bio, Inc.), according to the manufacturer’s instructions. The RT-PCR protocol was: one cycle at 50°C for 30 min; 2 min at 94°C; 30 cycles of 30 s at 94°C, 30 s at 58°C and 1 min at 72°C.

### Elimination of dsRNAs from Ath1

To obtain a virus-free line of Ath1 for comparison with the virus-infected line, plugs of mycelium from the edge of an Ath1 virus-infected culture (grown on PDA plates at 37°C in the dark) were transferred to PDA plates containing 21 mg/mL cycloheximide (Sigma) and incubated in the dark for 1 week at 37°C. Single conidiospore isolates were grown on unamended PDA media for 3 days at 37°C in the dark, followed by a second round of cycloheximide treatment (as described above). Single spore isolates were then grown on unamended PDA media in the dark for 1 week at 37°C, sub-cultured twice and then tested for the presence of virus.

Internal Transcribed Spacer (ITS) regions of the naturally virus infected and the generated virus free cultures were PCR amplified using ITS1F forward primer 5`-CTTGGTCATTTAGAGGAAGTAA-3`and ITS4 reverse primer 5`-TCCTCCGCTTATTGATATGC-3`[41] and sequenced. DNA was extracted using a ZR Fungal/Bacterial DNA Microprep kit (Zymo Research) according to the manufacturer’s protocol. PCR amplification was performed in a 20 μl reaction volume containing 12.2 μl ultrapure water (Invitrogen), 2 μl 10X AmpliTaq DNA Polymerase buffer, 2 μl 25 mM MgCl2, 0.4 μl 10 mM DNTPs mix (Life technologies)), 0.5 μl of 10 μM of each forward and reverse primers, 0.2 μl Dimethyl sulfoxide (DMSO), 0.2 μl AmpliTaq DNA Polymerase (Life technologies) and 2 μl DNA sample. The amplification programme was as follows: an initial denaturation for 3 min at 94°C; 40 cycles of 94°C for 15 s, 52°C for 45 s, and 72°C for 1 min, followed by a final extension step at 72°C for 6 min. Following PCR, the 20 μl DNA product was electrophoresed, visualized, purified, cloned and sequenced as described above.

### Virus impact on *Aspergillus* sporulation at 37°C and 20°C

Sporulation rate comparisons were conducted at 37°C, to represent human body temperature, and 20°C to represent an environmental temperature. Five virus-free cultures (obtained from virus-infected cultures using cycloheximide treatment) and five virus-infected cultures were grown from single spores and inoculated at the edge of PDA plates (9 cm Ø) and incubated in the dark until the mycelium reached the opposite side of the plate. Spores were harvested from the plates by washing twice with 20 mL 0.05% Tween 80 and filtering through cheesecloth into a 50-mL Falcon tube. The spores were collected by centrifugation at 8000 x g for 10 min and resuspended in 10 mL distilled water before being counted in a Neubauer chamber. For data analysis, an independent samples t-test was performed using SPSS version 21 (IBM SPSS statistics).

### Virus incidence in ascospores

To separate ascospores from possible contamination by conidia, prior to testing for virus infection, a preliminary heat-treatment experiment was extrapolated from O'Gorman et al. [42] and Girardin et al. [43] and performed to determine the temperature at which conidia were killed and ascospores survived. Cleistothecia (containing asci and ascospores) were picked individually from virus-infected Ath1 cultures using a sterile needle and gently dipped in a few drops of sterile distilled water. The cleistothecia were transferred individually to 2-mL Eppendorf tubes, each containing 0.5 mL 0.05% Tween 80 and some 0.1-mm silica beads. Cleistothecia were disrupted using TissueLyser II (Qiagen) at the highest frequency (30 Hz) for 1 min, and then aliquots of 100 μL each were pipetted into 0.2-mL PCR tubes. The samples were then heated to 65°C for 15 min, which had previously been determined to inactivate the asexual conidia but not ascospores [44].

One hundred ascospores were germinated individually on PDA plates for one day at 37°C in the dark and individual germlings transferred to 250-mL flasks containing 100 mL YPD and incubated at 37°C for 2 days on an orbital shaker at 180 rpm. AthCV1-specific one-step RT-PCR was conducted on the resultant mycelium to test for virus presence, as described above.

### Growth rate comparison of virus-infected and virus-free Ath1 at 20°C and 37°C

Virus-free and virus-infected cultures were grown for 7 days on PDA in the dark. To compare linear growth, five replicate virus-free and virus-infected single spore isolates were individually inoculated at the edge of PDA plates and grown at 37°C for 6 days and at 20°C for 15 days in the dark. The growth was measured every 24 h and at the completion of the experiment cultures were tested for the presence of the virus using RT-PCR as described above. To compare biomass production, five virus-free and virus-infected single spore isolates were grown at 37°C for 4 days and at 20°C for 15 days in the dark, and five replicate plugs of the resultant mycelium individually transferred to conical flasks containing 200 mL YPD and incubated on a shaking incubator at 180 rpm in the dark. Mycelium was collected by vacuum filtration; 100 mg of each sample being retained for virus testing (using one-step RT-PCR as described above) and the remainder dried at 90°C for 72 h before weighing. An independent samples t-test was performed using SPSS version 21 (IBM SPSS statistics) for data analysis.

### Presence of AthCV1 in phenotypically different sectors within fungal cultures

Formation of sectors with different growth phenotypes was frequently observed in the virus-infected Ath1 cultures. These sectors were either smooth white cottony, or with rough surface texture. Three plugs of mycelium (~1 cm^2^) from each sector were tested for the presence of the virus by RT-PCR using virus-specific primer pairs as described above.

### Protoplast preparation and virus reintroduction into virus-free Ath1

Since the virus-free culture of Ath1 did not reproduce sexually, it was important to determine whether the virus-free culture would produce sexual spores when it was transfected with the virus. Protoplasts were prepared according to the method of Kohn et al. [45] with modifications as follows: a virus-free single spore isolate was grown on a PDA plate for 24 h at 37°C in the dark, the whole mycelium transferred to a 500-mL conical flask containing 200 mL YPD and incubated for 1 day at 37°C on a shaking incubator at 180 rpm in the dark. A 5-g sample of mycelium was washed once with sterile distilled water and once with protoplast buffer (0.8 M MgSO_4_.7H_2_O, 0.2M C_6_H_5_Na_3_O_7_.2H_2_O, pH 5.5). The mycelium was then coarsely cut with a sterile blade in a glass beaker and transferred into a 150-mL flask containing 17 mL protoplast buffer. Then 0.2 g of Lysing Enzymes from *Trichoderma harzianum* (Sigma-Aldrich) was dissolved in 3 mL Novozyme buffer (1 M sorbitol, 50 mM sodium citrate, pH 5.8), filter sterilised through a 0.40-μm strainer and added to the mycelial suspension, followed by incubation in a shaking incubator at 85 rpm for 4 h at 28°C. Protoplast formation was checked every 20 min, starting from 60 min, and after 4 h the protoplasts were passed through a 75-μm strainer and collected in a 50-mL tube. A 30-mL volume of KC buffer (0.6 M KCl and 50 mM CaCl_2_) was immediately added to the protoplast suspension and the mixture centrifuged at 4000 x g for 10 min. The protoplast pellet was then washed twice with 10 mL STC (1M sorbitol, 50 mM Tris, pH 8, and 50 mM CaCl_2_.2H_2_O) at 4000 x g for 10 min before being resuspended in 0.5 mL STC and kept on ice.

Transfection was conducted as described by Rollins [46] with modifications as follows: 200 μL of purified virus particle suspension, extracted from 10 g of mycelium, was mixed with 5 μL 0.05 mM spermidine (Sigma-Aldrich). In a separate sterile microcentrifuge tube, 130 μL PEG 4000 (60% in water) was mixed with 70 μL KTC (1.8 M KCl, 150 mM Tris pH 8, 150 mM CaCl_2_) and added to the spermidine and virus particles suspension. A 200-μL aliquot of the protoplast suspension was then added to the virus suspension and mixed by twirling the tube for 10 s before it was incubated on ice for 30 min. Following incubation, 200 μL of PEG 4000 was mixed with 100 μL KTC in a separate tube, added to the previous suspension and gently twirled again and incubated at room temperature for 20 min. Following incubation, 40-μL aliquots of the mixture were added to 5 mL warm agar medium (Stabilized Minimal Medium (SMM) containing 0.7% agar), gently mixed and immediately spread on SMM plates which were sealed with Parafilm and incubated at 37°C [47]. Individual colonies were picked off, grown on fresh PDA plates, and sub-cultured three times (at weekly intervals) then checked for the presence of the virus by RT-PCR. For negative controls, three plates were spread with a protoplast suspension lacking virus particles to test protoplast viability; three plates were spread with no protoplasts in the transfection suspension to test for possible mycelial contamination in the virus particle suspension; and three plates containing only SMM media were included as a general contamination check.

### Infection stability in virus-transfected and naturally infected cultures

When grown on solid media, the naturally infected Ath1 culture usually exhibited a rough texture from which white cottony ascospore-free sectors repeatedly emerged. When subcultured, the white cottony ascospore-free sectors retained this phenotype and did not produce any sectors. Similarly, virus-transfected lines showed only the white cottony ascospore-free morphology. To try to explain these differences, the different phenotypes were tested for the virus over time to determine the presence and persistence of virus infection. Ten serial sub-cultures (each grown for one week) were made from each phenotype and total RNA extraction, and virus testing performed by RT-PCR, as previously described, using virus-specific primer pairs as follows: dsRNA1, forward 5`-AGCTCAGTCATCGAGATAGC-3`, reverse 5`-TCTATGTCGGGTCCCTCTAT-3`; dsRNA2, forward 5`-CGAGTGTGAGGCATCAAAGC-3`, reverse 5`-TGTCGCATGATGCATATAATTGGG-3`; dsRNA3, forward 5`-TGCTTTCTTCAACCACTTCC-3`, reverse 5`-TGAGTACTCGCACACGTC-3`; dsRNA4, forward 5`-TTGGCTGTACGTTGGCGATG -3`, reverse 5`-TGAATGGGTCCAGCCACTC -3`. Electrophoresis and DNA visualization was conducted as described previously.

## Results

### Presence of dsRNAs and virus particles in Ath1

**Fig 1:**
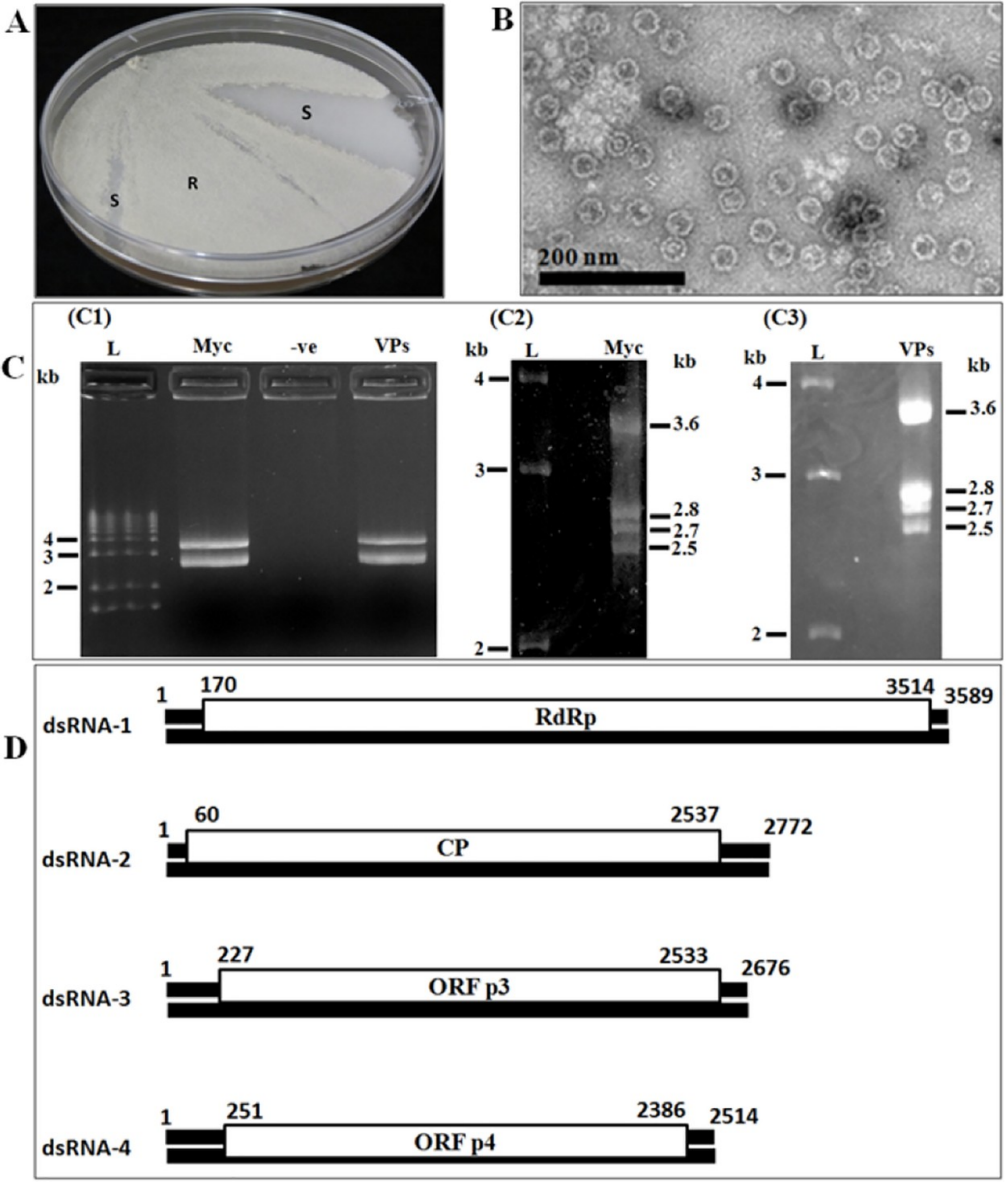
**(A). Sectoring phenotype in Ath1 virus infected cultures growing on PDA;** Soft white cottony texture (S) emerged from creamy rough granulated texture (R). **(B):** Negatively stained 35 nm virus particles from Ath1 negatively stained with 2% aqueous uranyl acetate, pH 4. **(C):** C1, dsRNAs extracted from mycelia of Ath1 and purified virus particles (1% agarose, 90 v for 45 min). L: 1 kb plus DNA ladder, Myc = dsRNA extracted from Ath1 mycelia, -ve = negative control extraction from dsRNA free *A*. *fumigatus*, VPs = dsRNA extracted from virus particles associated with Ath1; C2, separation of dsRNAs extracted from Ath1 mycelia (Myc) (1.5% agarose gel for 19 h at 80 v), L = 1 kb plus DNA ladder; C3, dsRNA extracted from virus particle associated with Ath1 (VPs), L: 1 kb plus DNA ladder. **(D):** Genome organization of AthCV1. The genome consists of four dsRNA segments, each of which is monocistronic. DsRNA1 encoded putative RdRP, dsRNA2 encoded CP, each of dsRNA3 and dsRNA4 encodes for HPs.

Initial electrophoresis of dsRNAs from Ath1 (both mycelium and virus particle) in 1% agarose gel at 90 V for 45 min showed the presence of two dsRNAs (~3600 bp and ~2700 bp), but subsequent electrophoresis in 1.5% agarose gel for 19 h at 80 V separated the lower band into three bands of 2.5, 2.68 and 2.7 kb (Fig 1C). Homogenised mycelium of Ath1 contained a high concentration of 35 nm isometric particles (Fig 1B).

### Molecular characterization of virus dsRNA genome

Partial genome sequence obtained by 454 sequencing was confirmed and extended by cloning and Sanger sequencing to obtain the complete nucleotide sequences of the four dsRNA segments of the virus and the sequences deposited in GenBank: dsRNA1 3589 nt (MF045841), dsRNA2 2772 nt (MF045842), dsRNA3 2676 nt (MF045843) and dsRNA4 2514 nt (MF045844). Each dsRNA segment contains a single open reading frame and 5`and 3`-UTRs (Fig 1D). DsRNA1 encodes a putative RdRP (1114 aa), dsRNA2 encodes a putative CP (825 aa), dsRNA3 and dsRNA4 encode proteins (768 & 711 aa, respectively) of unknown function. All four dsRNAs contain a highly conserved 20 nt sequence at the 5`terminus and also share some similarity in their 3`termini. The 5`-UTR of the four dsRNAs share a highly conserved sequence stretch U(/C)GCAAAAAAGAAGU(/A)AAAGGGG(/C), while the 3`-UTR of all four dsRNAs end with UGU. The CAA repeat region found at the 5`terminus of all four dsRNA genome segments of the type member of the genus *Chrysovirus* does not exist in any of the four dsRNA elements of AthCV1. The closest sequences in GenBank were the four dsRNAs of *Penicillium janczewskii chrysovirus* 2 (Table 1).

**Table 1:** Properties of AthCV1 dsRNA genome segments.

Genome segment	Accession number	Total length (nt)	5’ UTR (nt)	3’ UTR (nt)	Encoded protein (aa)	Most similar GenBank accession*	Identity % (aa)	E-value
dsRNA1	MF045841	3589	169	75	1114	PjCV2 (RdRP) ALO50149	60	0.0
dsRNA2	MF045842	2772	59	235	825	PjCV2 (CP) ALO50150	49	0.0
dsRNA3	MF045843	2676	226	143	768	PjCV2 (HP) ALO50151	41	1e-143
dsRNA4	MF045844	2514	250	128	711	PjCV2 (HP) ALO50152	46	2e-158

* *Penicillium janczewskii chrysovirus* 2

**Fig 2:**
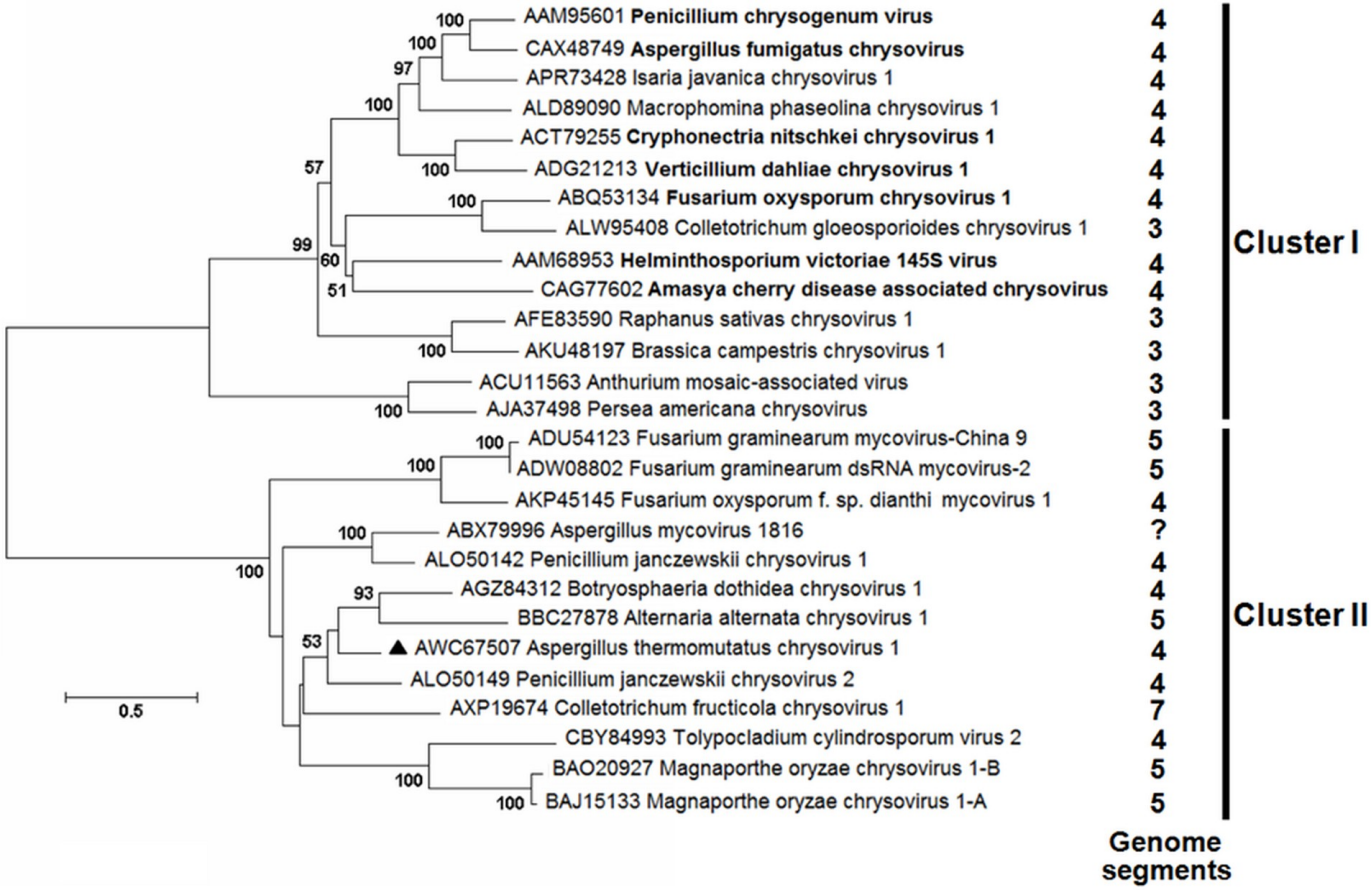
Phylogenetic analysis of *A*. *thermomutatus* chrysovirus 1 and its closely related members of the family *Chrysoviridae* and unclassified viruses based on amino acid sequences of their RdRPs. The amino acid sequences were aligned using the program MUSCLE [38]. A neighbour-joining phylogenetic tree [39] constructed from this alignment for distance calculated with a Poisson model and a gamma distribution of rates between sites using the program MEGA 6 [40]. Bootstrap values based on 1000 replicates. Cluster I contains viruses with 4 or 3 genome segments (classified members of the genus Chrysovirus are in bold font). Cluster II contains 4, 5 or 7 genome segments.

A phylogenetic comparison of the translated amino acid sequences from the RdRP, encoded by dsRNA1, with those of 26 other chrysoviruses (Fig 2) placed the *A*. *thermomutatus* virus in the same monophylogenetic clade with *Botryosphaeria dothidea chrysovirus* 1 [48], *Alternaria alternata chrysovirus* 1 [49] and *Penicillium janczewskii chrysovirus* 2 [50] with the latter being the closest descendent. The closest outgroup to this clade (Fig 2) consisted of *Colletotrichum fructicola chrysovirus* 1 [51]. Sequence similarity of the 5`and 3`termini of the four dsRNA genome segments were obtained. Proposed secondary structures for the 5`and 3`-UTRs of the positive strand of dsRNA1 were predicted to fold into stable stem-loop structures with a minimum free energy of -53 and -35.7 kcal/mol, respectively S1 Fig.

### Elimination of virus-dsRNA from Ath1

**Fig 3:**
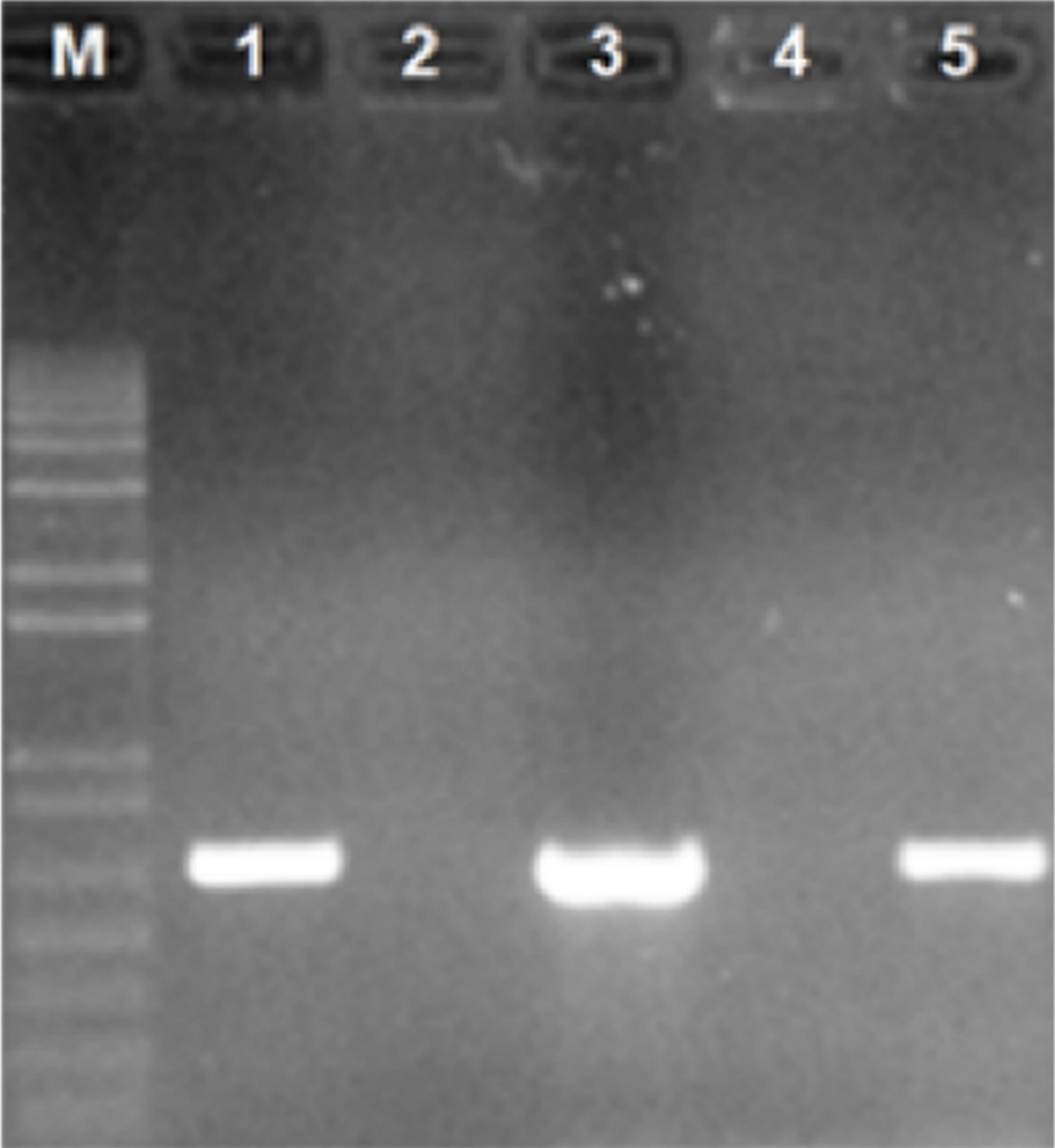
Successful elimination of AthCV1 with cycloheximide (PCR confirmation): lane M = 1 kb plus DNA ladder, lane 1 = ITS1 & ITS2 DNA band confirms competence of PCR in virus-free culture, lane 2 = ITS1 & ITS2 negative PCR control (RNA replaced with ultrapure water), lane 3 = virus-specific band in virus-infected line used as +ve control, lane 4 = No virus-specific band in virus-free line, lane 5 = ITS DNA band confirms competence of PCR in virus-infected line.

PCR screening and agarose gel electrophoresis of dsRNA revealed that a virus-free culture was successfully obtained (Fig 3) following culture on PDA media containing 21 mg/mL cycloheximide. Colony morphology of the virus-free culture was white cottony and ascospore-free, while the virus-infected culture was creamy, rough and ascospore-rich with the emergence of white cottony ascospore-free sectors (Fig 1A). ITS sequences (ITS1 & ITS2) obtained from the Ath1 virus infected culture (accession no: MK111646) and from the derived virus free culture (accession no: MK111645) were identical with the closest BLASTn hit (99% identity, accession no: AF459729) being the ITS sequence of *Neosartorya pseudofischeri* isolate NRRL (teleomorph of *Aspergillus thermomutatus*). Sequences of ITS 1 and 2 regions have been reported to identify Aspergillus species [52, 53].

### Viral impact on Ath1 growth and sporulation at 37°C and 20°C

The effects of viral infection and temperature on the growth and sporulation of Ath1 are summarized in Table 2. The virus-free culture line produced no ascospores at either 20°C or 37°C, whereas the virus-infected culture line produced a large number of ascospores at both temperatures, with a two-fold increase at 37°C. At 20°C conidiation was significantly decreased (*p*<0.05) in the virus-infected culture compared with that in cultures at 37°C where conidial production was ten-fold greater than in the virus-free culture. There was no significant difference (*p*<0.05) in the rate of mycelial growth or mycelial dry weight between virus-negative and virus-positive Ath1 cultures at either 20°C or 37°C.

**Table 2:** Effects of AthCV1 on growth and sporulation of Ath1 in culture.

Property	Cultured @ 20°C			Cultured @ 37°C		
	virus-free	infected	significant difference (*p*<0.05)	virus-free	infected	significant difference (*p*<0.05)
Conidia production (per plate)	2.4 X 10^6^	2.1 X 10^4^	decrease	2.1 X 10^4^	2.04 X 10^6^	increase
Ascospore production (per plate)	0	3.6 X 10^5^	Increase	0	7.2 X 10^5^	increase
Linear growth (mm)	76	75	NS	74	75	NS
Mycelial dry wt (mg)	1135	1132	NS	1249	1276	NS
Pigmentation and sector formation	Entire culture white & cottony, ascospore-free with no sectors	white & cottony areas ascospore-free, creamy & rough sectors ascospore-rich		Entire culture white & cottony, ascospore-free with no sectors	white & cottony areas ascospore-free, creamy & rough sectors ascospore-rich	

### Infection stability of Ath1 in cultures (a) naturally infected with AthCV1 and (b) *in vitro* transfected with AthCV1

RT-PCR screening detected AthCV1 in both the creamy rough ascospore-rich and the white cottony ascospore-free sectors of both the initial cultures of naturally infected Ath1 and after four successive subcultures. However, after ten successive weekly subcultures the virus was detected only in the cultures’ originating from and still expressing the creamy rough ascospore-rich phenotype. Similarly, cultures transfected with AthCV1, which showed the white cottony ascospore-free phenotype, tested positive for AthCV1 after four subcultures but negative after ten subcultures.

### Virus incidence in ascospores

RT-PCR screening for AthCV1 detected the virus in 37% of 100 single ascospore cultures. Although the ascospore isolation method included a heat treatment of 65°C for 15 min, this is unlikely to have inactivated a dsRNA virus.

## Discussion

Unusually for *Aspergillus* species in culture, a clinical isolate of Ath1 was observed to produce few conidia and large numbers of cleistothecia. In addition, the cultures produced sectors exhibiting two different phenotypes; a creamy rough granulated sector with conidia and ascospores, and a white cottony sector producing conidia only. These cultures were found to be infected with a chrysovirus, closely related to *Penicillium janczewskii chrysovirus* 2 [50] and *Botryosphaeria dothidea chrysovirus* 1, the latter being reported to have a hypovirulent impact on its fungal host and to cause sector formation in the infected cultures [48].

Chrysoviruses are non-enveloped isometric particles, with a multipartite genome of four unrelated monocistronic linear dsRNA segments. The type member of the genus *Chrysovirus* is *Penicillium chrysogenum virus* (PcV) in which dsRNA1 codes for the RdRP (1117 aa), dsRNA2 codes for the CP (982 aa), while dsRNA3 and dsRNA4 code for hypothetical proteins (912 aa and 847 aa, respectively) [54]. The 5`-UTRs are relatively long (140–400 nt) and have the potential to form extensive secondary structures. Chrysoviruses appear to be common in *Aspergillus* spp., including *A*. *foetidus* [11], *A*. *niger* [12], *A*. *flavus* [14] and most recently, *A*. *fumigatus* [19, 17].

**Fig 4:**
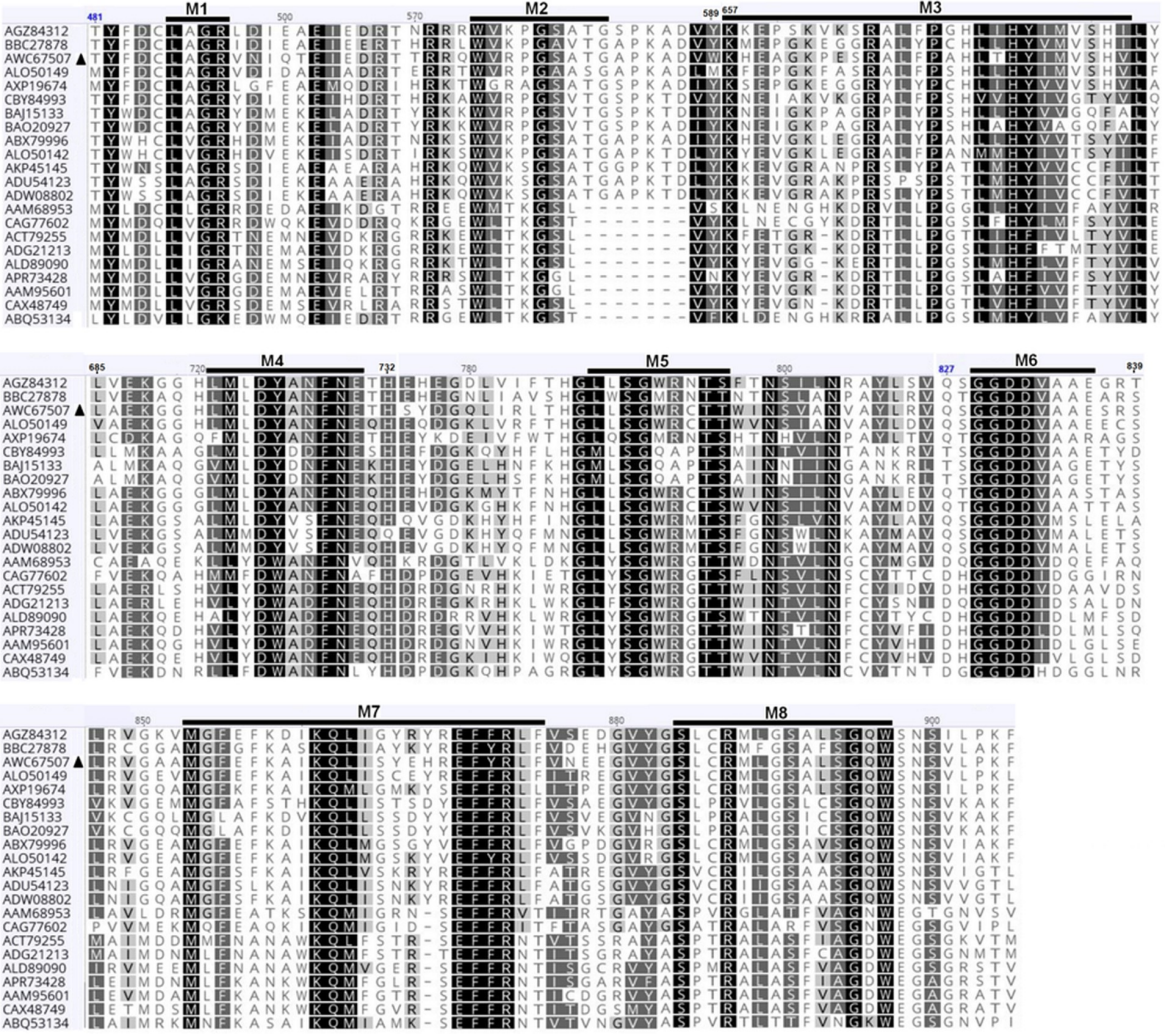
Alignment of amino acid sequences of the putative RdRP of *A*. *thermomutatus* chrysovirus 1 (AthCV1) with those of closely related chrysoviruses. The eight conserved RdRP motifs characteristic of dsRNA mycoviruses are referred to as M1-M8. Shading: black, 100% amino acid similarity; dark gray, 80–99% amino acid similarity; gray, 60–79% amino acid similarity; white, less than 60% amino acid similarity. Virus sequences are as follows: AGZ84312: RdRP aa sequence of *Botryosphaeria dothidea chrysovirus* 1 [48], BBC27878: RdRP aa sequence of *Alternaria alternata chrysovirus* 1 [49], AWC67507: RdRP aa sequence of AthCV1, ALO50149: RdRP aa sequence of *Penicillium janczewskii chrysovirus* 2 [50], AXP19674: RdRP aa sequence of *Colletotrichum fructicola chrysovirus* 1 [51], CBY84993: RdRP aa sequence of *Tolypocladium cylindrosporum virus* 2 [58], BAJ15133: RdRP aa sequence of *Magnaporthe oryzae chrysovirus* 1-A [59], BAO20927: RdRP aa sequence of *Magnaporthe oryzae chrysovirus* 1-B [60], ABX79996: RdRP aa sequence of *Aspergillus mycovirus* 1816 [61], ALO50142: RdRP aa sequence of *Penicillium janczewskii chrysovirus* 1 [50], AKP45145: RdRP aa sequence of *Fusarium oxysporum f*. *sp*. *dianthi virus* [62], ADU54123: RdRP aa sequence of *Fusarium graminearum mycovirus-China* 9 [63], ADW08802: RdRP aa sequence of *Fusarium graminearum dsRNA mycovirus*-2 [64], AAM68953: RdRP aa sequence of *Helminthosporium victoriae* 145S *virus* [65], CAG77602: RdRP aa sequence of *Amasya cherry disease associated chrysovirus* [66], ACT79255: RdRP aa sequence of *Cryphonectria nitschkei chrysovirus* 1[67], ADG21213: RdRP aa sequence of *Verticillium dahliae chrysovirus* 1 [68], ALD89090: RdRP aa sequence of *Macrophomina phaseolina chrysovirus* 1 [69], APR73428: RdRP aa sequence of *Isaria javanica chrysovirus* 1 [70], AAM95601: RdRP aa sequence of *Penicillium chrysogenum virus* [71], CAX48749: RdRP aa sequence of *A*. *fumigatus chrysovirus* [19], ABQ53134: RdRP aa sequence of *Fusarium oxysporum chrysovirus* 1 [72].

**Fig 5:**
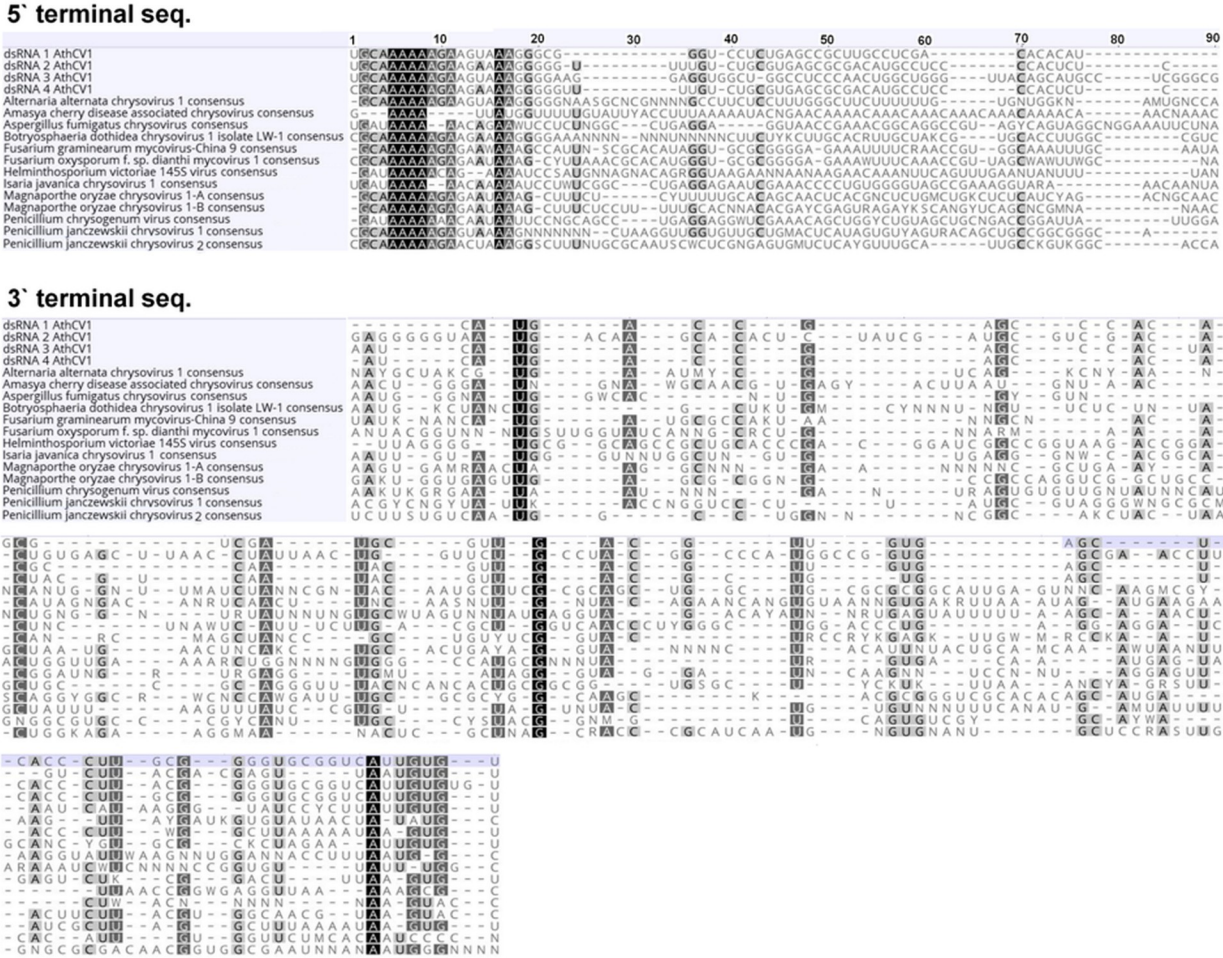
Multiple sequence alignments of the 5′- and 3′-terminal regions of the coding strands of the four individual AthCV1 dsRNA segments and consensus sequences of the 5′- and 3′-terminal regions of the genomes of other closely related chrysoviruses. Shading: black, 100% nucleotide identity; gray, 80–99% nucleotide identity; light gray, 60–79% nucleotide identity.

The virus isolated from Ath1 is the first virus described from *A*. *thermomutatus*. It exhibits all the common characteristics of chrysoviruses and is provisionally named *A*. *thermomutatus chrysovirus* 1 (AthCV1). The ~35 nm isometric particles encapsidate four dsRNA segments (dsRNA1 = 3589 nt, dsRNA2 = 2772 nt, dsRNA3 = 2676 nt, dsRNA4 = 2514 nt), with amino acid sequence similarity to known chrysoviruses (Fig 4, Table 3). Both 5`and 3`-UTRs of the four AthCV1 dsRNAs contain highly similar internal sequences and also show high similarity to the 5’ end of other chrysoviruses (Fig 5). In common with *Penicillium janczewskii chrysovirus* 2 (PjCV2) [50], the most similar chrysovirus, AthCV1 does not contain the (CAA)n repeats found in PcV, the type member of the genus *Chrysovirus* [55, 56, 57].

**Table 3:** Pairwise comparison of ORF1 (RdRP) between AthCV1 and its closely related chrysoviruses.

AthCV1	Virus name	Viral ORF	Length aa	Identity %	Similarity %	Ref.
ORF1	*Penicillium janczewskii chrysovirus* 2	ORF1	1119	60	73	[50]
	*Botryosphaeria dothidea chrysovirus* 1	ORF1	1116	56	72	[48]
	*Alternaria alternata chrysovirus* 1	ORF1	1119	55	70	[49]
	Colletotrichum fructicola chrysovirus 1	ORF1	1132	48	63	[51]
	*Penicillium janczewskii chrysovirus* 1	ORF1	1126	44	62	[50]
	Aspergillus mycovirus 1816	ORF1	1084	43	60	[61]
	*Tolypocladium cylindrosporum virus* 2	ORF1	1132	41	56	[58]
	*Magnaporthe oryzae chrysovirus* 1-A	ORF1	1127	38	54	[59]
	*Magnaporthe oryzae chrysovirus* 1-B	ORF1	1127	38	54	[60]
	*Fusarium oxysporum f*. *sp*. *dianthi mycovirus* 1	ORF1	1139	36	52	[73]
	*Fusarium graminearum dsRNA mycovirus*-2	ORF1	1137	35	52	[64]
	*Fusarium graminearum mycovirus-China* 9	ORF1	1137	35	52	[63]
	Amasya cherry disease associated chrysovirus	ORF1	1087	22	39	[66]
	*Helminthosporium victoriae* 145S *virus*	ORF1	1086	21	38	[65]
	Anthurium mosaic-associated virus	ORF1	1099	21	37	[74]
	*Macrophomina phaseolina chrysovirus* 1	ORF1	1105	20	38	[69]
	Colletotrichum gloeosporioides chrysovirus 1	ORF1	1088	20	37	[75]
	*Penicillium chrysogenum virus*	ORF1	1117	20	36	[71]
	*Isaria javanica chrysovirus* 1	ORF1	1117	20	36	[70]
	Verticillium dahliae chrysovirus 1	ORF1	1108	20	36	[68]
	Persea americana chrysovirus	ORF1	1093	20	36	[76]
	Brassica campestris chrysovirus 1	ORF1	1146	20	35	[77]
	*Aspergillus fumigatus chrysovirus*	ORF1	1114	20	35	[78]
	Raphanus sativas chrysovirus 1	ORF1	1138	19	35	[79]
	*Cryphonectria nitschkei chrysovirus* 1	ORF1	962	19	33	[67]
	*Fusarium oxysporum chrysovirus* 1	ORF1	858	17	30	[72]

Sector formation in cultures of Ath1 appeared to be associated with the presence of AthCV1. Sector formation in virus-infected fungal cultures, has previously been observed in a range of fungal species, including *Aspergillus* spp. [3, 46]. Conidia-free sectors of *A*. *fumigatus* were found to be infected with a chrysovirus, a partitivirus [16], a tetramycovirus [20], and aconidial sectors have also been observed in virus-infected *A*. *niger* cultures [6]. In AthCV1-infected Ath1, the presence of the virus appeared to trigger a switch from conidiospore production to ascospore production, but within these cultures white cottony sectors producing conidia only spontaneously appear. While AthCV1 could initially be detected in the white cottony sectors, it could not be detected after ten serial subcultures, suggesting the white cottony sectors are the result of low virus titre.

The vertical transmission of mycoviruses through conidia is often highly efficient and can be close to 100% in some cases [80]. In contrast, transmission via sexual spores is usually less efficient [81], although Varga et al. [8] reported that transmission of dsRNAs through the ascospores of *Neosartorya hiratsukae* (Anamorph: *Aspergillus hiratsukae*) was very efficient (percentage not given). Transmission of AthCV1 through the ascospores of Ath1 was relatively low (37%), thus providing a means for the fungus to escape from virus infection. The apparent ability of virus-infected cultures spontaneously to produce ascospore-free sectors that after multiple subcultures result in virus-free cultures, may also provide a means for the fungus to escape virus infection *in vivo*, and is worthy of further investigation.

The isolate Ath1 was the only isolate of its species being studied, within a biosafety cabinet, and using rigorous decontamination procedures to avoid contamination resulting in an extremely low chance of contamination. Regardless, it was considered important to double confirm the identity of the virus-free culture by sequencing the ITS 1 and ITS 2 regions as it has been previously used to identify *Aspergillus* species [82, 52, 53]. No cross-contamination was identified in virus-infected or virus-free lines.

The variable production of both asexual and sexual reproductive structures in virus-infected Ath1 cultures has implications for virus spread within the fungal population. The effect of AthCV1 on conidia production was dependent on temperature, with production of conidia in virus-infected cultures being significantly lower at 20°C and significantly higher at 37°C than in the virus-free cultures. It is uncertain whether this is unusual, as the majority of published studies report only the effects of mycoviruses on their host at a single temperature. However, these findings do highlight the importance of the virus-host-environment interaction.

Use of mycoviruses as biological control agents for fungi is a topic of wide interest, but an effective biological control agent requires consideration of multiple factors (host, virus and environment), as is well documented for chestnut blight control [80]. The first goal is finding a virus that is capable of inducing serious impact on its fungal host. Some tentative members of the genus *Chrysovirus*, such as *Botryosphaeria dothidea chrysovirus* 1 [48], *Magnaporthe oryzae chrysovirus* 1-A [59], *Magnaporthe oryzae chrysovirus* 1-B [60], *Agaricus bisporus virus* 1 [83], are known to decrease virulence or cause other phenotypic changes in their fungal hosts. In addition, *Aspergillus* mycovirus 1816 was reported as a probable suppressor of RNA silencing [61]. While AthCV1 had no significant effect on mycelial growth of Ath1 in culture, the low conidiation rate of virus-infected lines would presumably reduce the rate of spread of the fungus, leading to a reduced impact on hosts of the fungus.

A major constraint on the use of mycoviruses as biocontrol agents is that natural horizontal spread typically requires hyphal anastomosis, which is limited by genetically controlled hyphal incompatibility [84], thereby limiting transmission to other species and even within a species. Consequently the direct use of mycoviruses as biological control agents, especially in a clinical context, is very challenging. However, it is often possible to infect other fungal species experimentally (e.g. transfection with viral particles) to determine the effects on that new host [85] and whether these are less, more, or similar to the effects on the original host [86, 87]. Consequently it would be interesting to determine whether AthCV1 is capable of infecting other *Aspergillus* species and whether it could have significant effects on their biological properties, especially on sporulation. Even if it is impractical to use AthCV1 directly as a biocontrol agent, an understanding of the molecular nature of the effects caused by the virus may enable the development of approaches such as targeting gene expression by pharmaceuticals.

## Supporting information

S1 Fig Secondary structure proposed for the 5`-UTR (left) and 3`-UTR (right, two predicted structures) of the plus strand of AthCV1 dsRNA1 (putative RdRP). Minimum free energy is -53 and -35.7 kcal/mol for 5`and 3`termini respectively. Jop parameters: RNA at 37°C, Na+ = 1 M, Mg++ = 0 M, sequence type (linear), distance between paired bases (no limit).
(TIF)
