# Supplementary figures and images for: A novel chrysovirus from a clinical isolate of Aspergillus thermomutatus affects sporulation
Source: PLoS One. 2018 Dec 20;13(12):e0209443. doi: 10.1371/journal.pone.0209443 (PMC6301774; doi:10.1371/journal.pone.0209443)

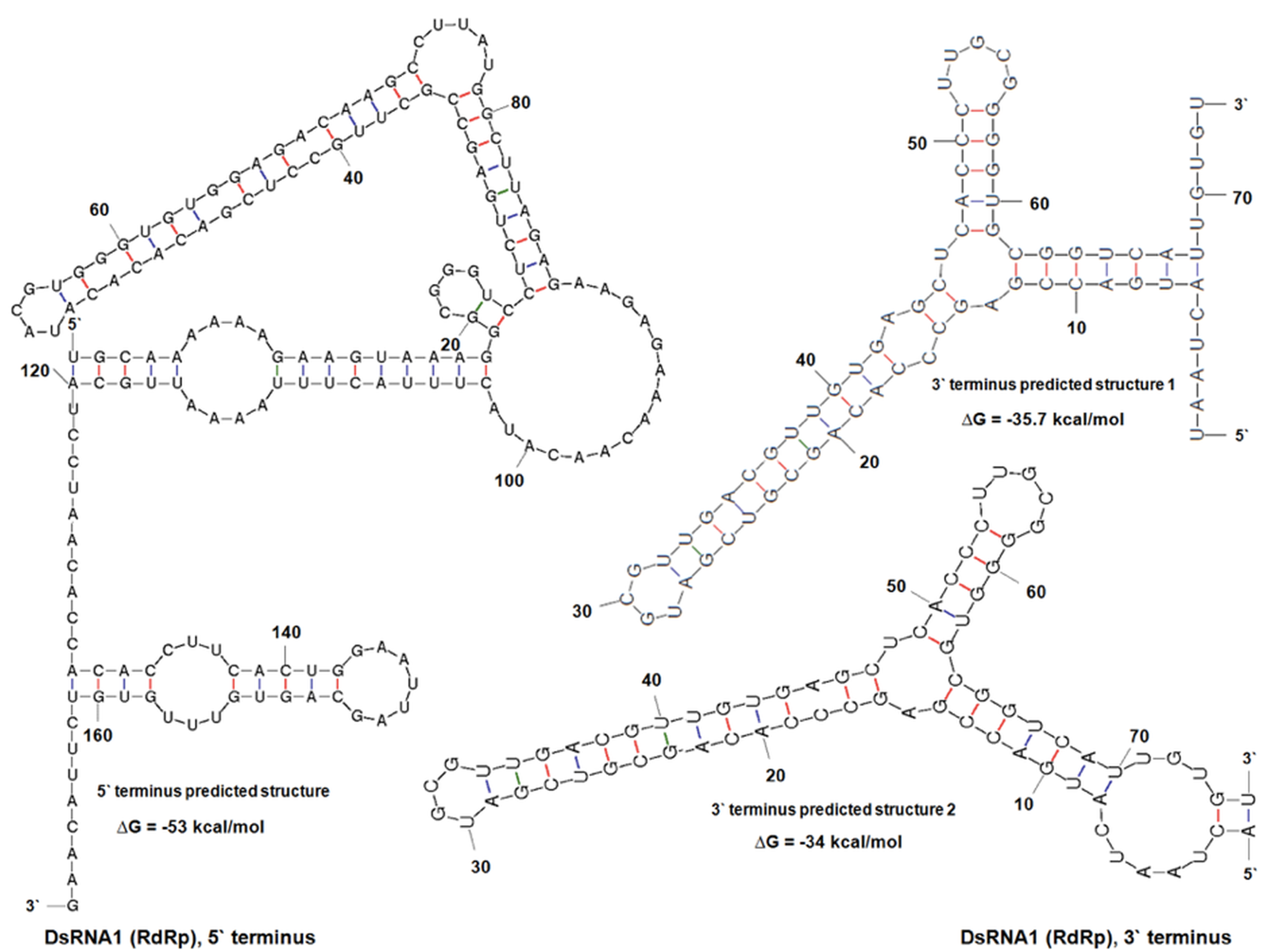

Supplement: S1 Fig — Secondary structure proposed for the 5`-UTR (left) and 3`-UTR (right, two predicted structures) of the plus strand of AthCV1 dsRNA1 (putative RdRP). Minimum free energy is -53 and -35.7 kcal/mol for 5`and 3`termini respectively. Jop parameters: RNA at 37°C, Na+ = 1 M, Mg++ = 0 M, sequence type (linear), distance between paired bases (no limit). (TIF) [file pone.0209443.s001.tif]
